# Long-Term Follow-Up of Pediatric Patients with Severe Postoperative Pulmonary Hypertension After Correction of Congenital Heart Defects

**DOI:** 10.1007/s00246-021-02794-9

**Published:** 2021-12-06

**Authors:** Lars Lindberg

**Affiliations:** 1 Institution of Clinical Science, Faculty of Medicine, Lund University, Lund, Sweden; 2 Lund University and Children’s Hospital, PICU, Skane University Hospital in Lund, 221 85 Lund, Sweden

**Keywords:** Congenital heart disease, Pulmonary hypertension, Pulmonary artery pressure, Pediatric cardiac surgery

## Abstract

The surgical repair of congenital heart defects in children with preoperative pulmonary hypertension (PH) is to varying degree associated with the occurrence of postoperative PH. The objective of this study was to follow up children with severe postoperative PH (pulmonary arterial/aortic pressure ratio ≥ 1.0) to evaluate if pulmonary arterial pressure spontaneously normalized or needed PH-targeting therapy and to identify potential high-risk diagnoses for bad outcome. Twenty-five children who developed clinically significant severe PH on at least three occasions postoperatively were included in the follow-up (20–24 years). Data from chart reviews, echocardiographic investigations, and questionnaires were obtained. Three children died within the first year after surgery. Three children were lost to follow-up. The remaining 17 children normalized their pulmonary arterial pressure without the use of PH-targeting drugs at any time during the follow-up. Two children had a remaining mild PH with moderate mitral valve insufficiency. All three children with bad outcome had combined cardiac lesions causing post-capillary pulmonary hypertension. Normalization of the pulmonary arterial pressure occurred in almost all children with severe postoperative PH, without any need of supplemental PH-targeting therapies. All children with bad outcome had diagnoses conformable with post-capillary PH making the use of PH-targeting therapies relatively contraindicated. These data emphasize the need to perform randomized, blinded trials on the use of PH-targeting drugs in children with postoperative PH before accepting it as an indication for routine treatment.

## Introduction

The impact of preoperative pulmonary hypertension (PH) on the outcome after correction of pediatric congenital cardiac defects has been a matter of discussion for a long time [1–3]. Some recognize PH in connection with pediatric cardiac surgery as an ominous sign and an increasing number of physicians advocate a preoperative catheterization to determine the pulmonary vascular resistance (PVR) in a child with preoperative PH before the child is accepted for surgical correction of the heart defect. However, a PVR level contraindicating cardiac surgery in children has not been defined. Some physicians even recommend the use of PH-targeting drugs both pre and postoperatively in children with preoperative PH to protect the children from the potential risk of develop right ventricular dysfunction [4, 5]. The fear and anxiety of postoperative PH have led to a dramatic increase in the use of pulmonary arterial dilating drugs, which have increased the cost of treatment [6, 7], but the scientific proof of improved outcome is lacking [5, 8].

It is therefore of interest to analyze if children with clinically significant postoperative severe PH following pediatric cardiac surgery need PH-targeting drugs to protect the right ventricle or to normalize their pulmonary arterial pressures. We have a unique cohort of 25 children who developed proven severe postoperative PH (pulmonary arterial/aortic pressure ratio, ≥ 1.0) in the era before phosphodiesterase 5 inhibitors and endothelin receptor antagonists were approved in Europe. The cohort was collected during a 5-year period (1994–1998) when we determined the incidence of clinically important postoperative PH in all children scheduled for open heart surgery [9]. A pulmonary artery catheter was placed in the main pulmonary artery through the right ventricular outflow tract in all children deemed at risk for postoperative PH; for example, children with unrestrictive VSD, complete AVSD, left-sided cardiac defect with high risk of PH, such as high left atrial pressure (> 18 mm Hg) or pulmonary venous stenosis, or children identified as having intraoperative PH (> ½ systemic pressure) measured by direct recording after the closure of the defects. The catheter guaranteed an accurate continuous measurement of the pulmonary arterial blood pressure in the postoperative period. The children had all been selected to be surgical candidates, since they had not developed signs of Eisenmenger’s syndrome according to the preoperative investigation.

The aim of this long-term follow-up analysis was to evaluate if children with severe postoperative PH used PH-targeting treatment (phosphodiesterase 5 inhibitors and/or endothelin receptor antagonists) to normalize their pulmonary arterial pressure in the long term. In addition, we identified high-risk diagnoses for bad outcome.

## Methods

### Study Patients and Data Collection

An Ethics Board decision (Dnr 2019–05,002) was requested and the need for patient consent was waived.

**Fig. 1:**
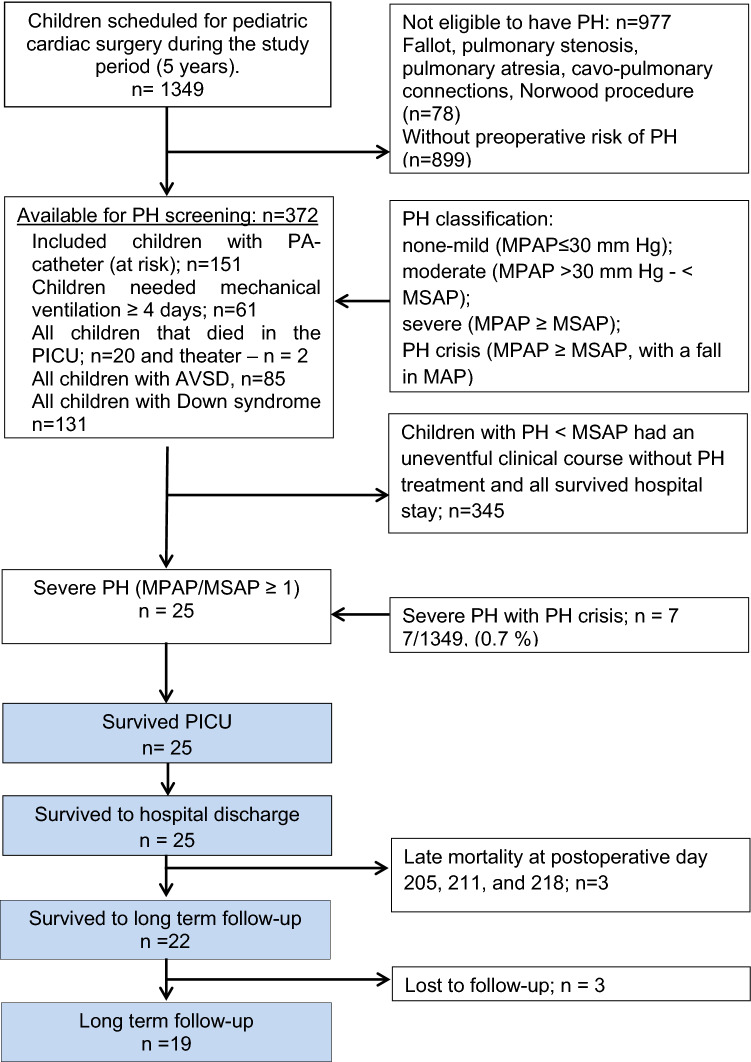
Flowchart showing numbers of patients at each stage of their course after surgery. *PH* Pulmonary hypertension, *PA* pulmonary arterial, *PICU* pediatric intensive care unit, *AVSD* atrioventricular septal defect, *MPAP* mean pulmonary arterial blood pressure, *MSAP* mean systemic arterial blood pressure, *MAP* mean arterial blood pressure

Twenty-five (*n* = 25) children (median age of 4.2 months, 0.03 – 59 months) who developed severe postoperative PH defined as a mean pulmonary arterial pressure equal to or exceeding mean systemic arterial pressure (pulmonary arterial/aortic pressure ratio, ≥ 1.0) on at least three occasions after pediatric cardiac surgery were included in the follow-up study. These 25 children were a subset of 1349 who underwent cardiac surgery at the Section for Pediatric Cardiac Surgery, Skane University Hospital, Lund University, Sweden, during a 5-year period (1994–1998) and were included in an earlier publication describing the incidence of different grades of PH at our clinic [9]. A summary of the number of children included in that study and the main findings—prior to this long-term follow-up study—is described in Fig. 1.

Children at risk of pulmonary hypertension, as described earlier, were routinely and prospectively equipped and monitored with a pulmonary arterial pressure catheter. There were 345 children with postoperative PH below systemic arterial pressure (pulmonary arterial/aortic pressure ratio, < 1.0; in this group there was no hospital mortality, no need for prolonged mechanical ventilation, and no echocardiographic signs of persistent PH that influenced postoperative course or treatment). PH crisis was defined as severe PH combined with a fall in systemic arterial pressure causing circulatory shock. Twenty-four of the 25 children with severe postoperative PH were identified from measurements by the pulmonary arterial catheters in place and one with transthoracic Doppler echocardiographic measurement.

The majority of the children had left-to-right shunt lesions (*n* = 18). Ten of the children had some variety of left heart obstruction (ranging from pulmonary vein stenosis to supra-annular mitral stenosis and unbalanced ventricle physiology) alone or in combination with left-to-right shunts and three were D-TGA (Table 1).

**Table 1:** Clinical characteristics of the studied children (*n* = 25) with perioperative severe pulmonary hypertension and their follow-up data

Age	Weight	Syndrome	Diagnosis	Cath/Ang	Operation	PICU	PHC	INO	PHDD	Postoperative UCG	Outcome	Medication
5 m	4.3	Down	C AVSD, Large unrestricted VSD	No	Two-patch technique	LV-failure	YES		No	OK	Survived	Levaxin
3 m	4.8	Down	C AVSD + MR + double orifice Large ASD and unrestricted VSD	No	Two-patch technique	MR		YES	No	OK	Survived	No
4 m	4.7	Down	C AVSD + MR Large unrestricted VSD	No	Two-patch technique				No	Min MR, No TR	Survived	No
16 m	8.3		C AVSD, Large ASD and moderately large VSD; PVRI 13.2 Wood U x m2; Qp/Qs 1.7/1	Yes	Two-patch technique				No	Min MI,TR pv 2.7 m/s, minimal residual VSD	Survived	No
4y	12.4		MR + MS (post endocarditis) PWCP 27 mm Hg, PAP 88/59, AP 94/67 mm Hg	Yes	Mitroflow 23				No	Reop MS, Min TR, EF reduced	Survived	Enalapril, Warfarin
13 m	7.6		Supravalv MS + parachute valve + VSD PSAP 70, SAP 70, LAP 16 mm Hg	Yes	Membrane res + commissurotomy	MR	YES		No	Min MR, TR peak vel 2.6 m/s	Survived	No
3y	12.6		MR + left pulmonary vein stenosis PVRI 7 Wood Unit x m2	Yes	Valvuloplasty of mitral valve		YES		No	Reop SAS, Min MR, MS, AS	Survived	No
4d	3.4		TAPVD + Hypoplastic LV + obstructive PV, Systemic RV pressure	Yes	Correction of TAPVD	RV-failure			No	Min TR, Normal PAP	Survived	No
2y	8.9		Supravalvular MS + VSD PCWP 20 mm Hg, PAP 54/23 mm Hg	Yes	Resection of membrane				No	Reop Coa, MR, TR with pv 3 m/s	Survived	Furosemide, Salbutamol
3 m	4.2		TGA + Large unrestrictive VSD	Yes (BAS)	Arterial switch + VSD closure	LV-failure	YES	YES	No	Stenosis in right PA	Survived	No
5 m	5.7	Down	C AVSD, Large unrestrictive VSD, MR	No	Two-patch technique				No	Mod MR, TR peak vel 3 m/s	Survived	
3 m	4.4	Down	C AVSD + PDA + CoA	No	Two-patch technique	LV-failure + MR			No	OK	Survived	Levaxin
1 m	3.9	Down	ASD + PH + VCS sin; SaO2 86–87%	No	ASD closure				No	OK	Survived	
3 m	3.8	VACTERL	TAPVD + 3 VSDs, PA – systemic pressure; Large unrestrictive VSD	Yes	TAPVD closure + VSD closure				No	OK	Survived	
9d	2.8		TGA	No	Arterial switch	Thrombosis VCS		YES	No	Min TR peak vel 2.3 m/s	Survived	Baclofen infusion
2y	8.3	Down	Unbalanced C AVSD, single papillary muscle, PH	No	Two-patch technique		YES		No	OK	Survived	No
6 m	6.2	Down	C AVSD, Large unrestrictive VSD	No	Two-patch technique	LV-failure			No	Min MR, no TR	Survived	No
3 m	6.2		Unbalanced C AVSD	No	Two-patch technique	RV-failure			No	Min MR and MS	Survived	No
6 m	7		TGA + VSD + Mitral straddling	Yes	Arterial switch + VSD closure				No	Moderate AR	Survived	No
13 m	7.8	Down	VSD, ASD, Qp/Qs 2.4:1	Yes	VSD and ASD closure						LTFU	
4 m	5.1		VSD, parachute Mitral valve	No	Dividing papillary muscle division, VSD closure					Reop × 3	LTFU	
3 m	4.9	Down	C AVSD, CoA PVRI 11.2 Wood Unit x m2; Qp/Qs 0.7:1	Yes	Two-patch technique						LTFU	
7w	4.6	Down	Hypoplastic LV and mitral valve + ASD	No	Exploration of mitral valve + ASD closure	Severe MR, prolonged vent	YES		No	Severe MR	POD 218	
4 m	5.8	Down	Unbalanced C AVSD	No	Two-patch technique	RV-failure, arrhythmia	YES		No	EF reduced	POD 205	
4 m	3.9		Hypoplastic LV and MS	No	Exploration of mitral valve + papillary muscle division	Inoperable, Severe MR			No	Severe MR	POD 211	

Age; *d* days, *w* weeks, *m* months, *y* years, *AR* Aortic regurgitation, *AS* Aortic stenosis, *ASD* atrial septal defect, *BAS* balloon atrial septostomy, *Cath/Ang* catheterization/angiography, *CoA* Coarctatio of aorta, *C AVSD* Complete atrioventricular septal defect, *dx* dexter, *EF* Ejection fraction, *Hg* Mercury, *INO* Inhaled Nitric oxide, *LTFU* Lost to follow-up, *LV* Left ventricle, *M* mitral, *Min* minimal, *MR* Mitral valve regurgitation, *MS* Mitral valve stenosis, *obstr* Obstruction, *OK* normal, *PAP* Pulmonary arterial pressure, *PSAP* Pulmonary systolic arterial pressure, *PCWP* pulmonary capillary wedge pressure, *PDA* Persistent ductus arteriosus, *PH* pulmonary hypertension, *PHC* Pulmonary hypertensive crisis, *PHDD* Pulmonary hypertensive dilating drugs, *PICU* pediatric intensive care unit, *POD* postoperative day, *pv* peak velocity, *PV* Pulmonary venous, *PVRI* pulmonary vascular resistance index, *Qp* pulmonary blood flow, *Qs* systemic blood flow, *Res.* Resection, *RV* Right ventricle, *SAP* systemic arterial pressure, *SAS* Subaortic stenosis, *TAPVD* Total anomalous pulmonary venous drainage, *TGA* Transposition of the great arteries, *TR* Tricuspid valve regurgitation, *UCG* Ultrasound cardiography, *VACTERL* Syndrome stands for Vertebral defects, Anal atresia, Cardiac defects, Tracheo-esophageal fistula, Renal anomalies, and Limb abnormalities; *VCS* Vena cava superior, *VSD* ventricle septal defect; Weight (kg)

Eleven children had preoperative cardiac catheterization and/or angiographic examination. PVRI was determined in three children and was found to be 13.2, 11.2, and 7 Wood units × m^2^, respectively, Table 1.

Primary data from chart reviews and available medical databases were retrieved. A questionnaire was constructed to receive data of the clinical course during the follow-up period after the primary surgical procedure. Need for reoperation, clinical condition, variables from echocardiographic investigations with particular focus on signs of PH, and use of any PH-targeting drugs during the follow-up time were requested. The questionnaire was sent to the referring physician and/or the physician who is or was responsible for the medical treatment.

### Study Outcome Measures

The outcome measures of this study were treatment with PH-targeting drugs, rate of mortality, and the prevalence of high-risk diagnoses related to severe PH. Mortality data were obtained from the Swedish National Civil Population Register, which by law has to register all mortality in Sweden within 24 h of death.

### Statistical Analysis

Data were analyzed with the STATISTICA for Windows software package (TIBCO software Inc. Palo Alto, CA 94,304, USA). Descriptive statistics are expressed as median and range.

## Results

The median age at the time of repair was 4.2 months, range 0.03 – 59 months (*n* = 25) for children who developed severe postoperative PH and the follow-up time was 20–24 years.

All children who developed repeated episodes of postoperative severe PH in the PICU, of which seven children also had pulmonary hypertensive crisis (PHC), survived to hospital discharge (25/25, 100%). Three children were given nitric oxide inhalation during PHC.

Of the 25 children, three children died within the first year at 205, 211, and 218 days, postoperatively. They all had a diagnosis of borderline hypoplastic left ventricle and/or significant mitral valve pathology, Table 1.

Of the remaining 22 patients, three patients were lost to follow-up. None of the three patients were reported as deceased according to the Swedish National Civil Population Register and no address was registered in Sweden indicating that they had left the country. Data were retrieved from the remaining 19 patients.

All remaining 19 patients survived the follow-up period. Echocardiographic findings showed that 36% of the children already had normalized their pulmonary arterial pressure at hospital discharge. Sixty-three percent left the hospital after the primary surgical correction with a significant remaining PH measured as a tricuspid valve regurgitation (TR) exceeding a peak velocity of 2.5 m/s, corresponding to a pulmonary arterial systolic pressure (PASP) of approximately ≥ 35 mmHg if central venous pressure was estimated to be ≤ 10 mm Hg. One child had a minimal residual VSD (the child with a preoperative PVRI of 13.2 Wood units x m^2^), but otherwise had an uneventful recovery.

None of the 19 patients received any kind of PH-targeting drugs (phosphodiesterase inhibitors, endothelin inhibitors, or inhaled vasodilators) during the follow-up period in the ward or after hospital discharge according to medical records or retrieved data, not even after PH-targeting drugs were approved in Europe. Seventeen of these children had normal pulmonary arterial pressure according to echocardiographic findings.

Two of the 19 patients had slightly elevated PH remaining, with a peak velocity across the tricuspid valve of 3 m/s, indicating a pressure gradient across the valve of approximately 36 mmHg. The echocardiographic investigations showed that both these patients had moderate mitral valve regurgitation.

Thirteen patients had normal neurological development postoperatively. Seven patients were diagnosed with syndromes (six patients with Down syndrome and one patient with VACTERL association).

One patient with TGA had suffered anoxic brain damage after an incident of cardiac arrest due to a ventricular arrhythmia 11 years after the surgical correction and was treated with baclofen, due to spasticity.

Five patients used medications, such as diuretics, warfarin, levothyroxine, ACE inhibitors, or baclofen (Table 1).

One patient had undergone resternotomy because of significant mitral valve stenosis and one a thoracotomy because of coarctatio aortae during the follow-up period. One had received a cerebral ventricular-peritoneal shunt due to hydrocephalus.

## Discussion

Most of the children with severe postoperative PH, defined as a mean pulmonary arterial pressure equal to or exceeding mean systemic arterial pressure (pulmonary arterial/aortic pressure ratio, ≥ 1.0), had shunt lesions. After the surgical correction, the pulmonary arterial pressure normalized in all children without the use of supplemental PH-targeting treatment, expect in two children with remaining moderate mitral regurgitation, consistent with post-capillary PH.

Several of the children had unrestrictive VSD, which by definition implies that the children had equilibrated blood pressures between the left and right ventricles, and bidirectional shunting through the VSD. This was verified by the preoperative echocardiographic investigation. It is difficult to distinguish children with preoperative PH and irreversible pulmonary vascular damage that are not compatible with survival after the surgical cardiac correction from those with PH and reversible pulmonary vascular pathology. Preoperative catheterization was performed in eleven children, in most cases for morphologic mapping. In three children with complete AVSD, PVRIs were determined in order to facilitate the decision for surgery. The PVRIs were quite high, ranging between 7 and 13.2 Wood units x m^2^, which uniformly would rule out corrective surgery. However, the decision to perform cardiac surgery proved successful in all three children, indicating that the PVR was indeed reversible. It also emphasized the findings by several authors that there is no association between preoperative PVRI and outcome [10, 11] and that the response to vasodilator testing showing reversibility has no prognostic implication, published in the 5th World Symposium on pulmonary hypertension in Nice, France, 2013 [11, 12].

All 25 children were deemed to be surgical candidates and were preoperatively viewed as candidates for surgical correction. However, three of the children with severe postoperative PH died during their first year of life and all three had hypoplastic left ventricles with two having hypoplastic mitral valves, as well. It can be argued that these children should have been corrected with a Fontan circulation, but there is always an intermediate zone, where the “hypoplastic” ventricle and/or the valves (mitral, aortic) are not small enough to disqualify the child for a biventricular correction. At the time of surgery, the preoperative decision to aim for biventricular circulations, in these children proved incorrect.

All of the remaining children who proved to have severe postoperative PH and did not have mitral regurgitation and survived the first postoperative year normalized their pulmonary pressure and showed no signs of remaining clinically important PH during the follow-up period. None of the children were on PH-targeting drugs—nor had they received such treatment during their postoperative course. These data indicate that preoperative changes that caused and increased in PVR were reversible and that the normalization and outcome were not dependent on PH-targeting drugs.

These findings agree with the review by Suesaovalaki et al. [13] who noticed that pulmonary arterial pressure and PVR normalized in all patients after surgical correction of left-to-right shunts regardless of the severity of the pulmonary vascular changes and that the therapies should be directed to improve right ventricular performance rather than using pulmonary vasodilating drugs [14].

Although the children had a variety of diagnoses, most of our children who developed severe postoperative PH had lesions with increased pulmonary blood flow and PH. The preoperative PVRI was only determined in three children and was shown to be increased. PH increases myogenic activity in the smooth muscle cells of the pulmonary vessels and thereby increases PVR to levels that shift the shunt from left to right to bidirectional. However, the increase in PVR simultaneously protects the pulmonary capillaries from the high pulmonary arterial pressure and high pulmonary capillary filtration pressure. A high pulmonary capillary filtration pressure may lead to pulmonary oedema. In the long run pulmonary vascular occlusive disease may occur and Eisenmenger’s syndrome may develop. The remodeling of the smooth muscle cells in the pulmonary arterial vessels caused by the preoperative PH results in a risk of clinically significant PH after the correction of the cardiac defects. The right ventricle has simultaneously been trained by the high pulmonary arterial blood pressure and would, therefore, potentially be better prepared to cope with a high right ventricular pressure after the correction, especially when the volume overload is eliminated and the wall tension in the ventricle consequently has been decreased. Our children were scheduled for surgery on the basis that the shunt was at most bidirectional and there was no consensus on what PVR definitively contraindicates surgery. The only treatable and curative solution for these children was correction and the goal for the preoperative investigation was to exclude that Eisenmenger’s syndrome had developed at time of diagnosis. Our best choice to guide postoperative care was to insert a catheter in the pulmonary truncus to monitor the pulmonary arterial blood pressure continuously.

PH less than systemic arterial pressure (pulmonary arterial/aortic pressure ratio, < 1.0) did not affect outcome, time of weaning from ventilator, clinical management, or time in the PICU. All these children had a clinical uneventful course in the cardiology ward before hospital discharge. Interestingly, children with postoperative PH less than systemic arterial pressure are often included in studies to test the effect of PH-dilating drugs, like inhaled nitric oxide and sildenafil [15–18], and no study showed any conclusive evidence that these treatments are of significant benefit. The drugs may decrease pulmonary arterial pressure and PVR [19], but had no clear impact on postoperative care or outcome. It seems that as long as the right ventricular pressure is less than left ventricular pressure, recovery of the right ventricular function after correction of the shunt is quite prompt. PH-dilating drugs may decrease pulmonary arterial pressure and PVR but were not needed as long as the right ventricle could cope with its pressure load.

Pulmonary arterial/aortic blood pressure ratio ≥ 1.0 postoperatively indicates a remaining significantly increased PVR, during the episodes of severe PH, since the pulmonary and systemic blood flows after the correction and closure of the shunts are almost similar. However, even in this cohort of children with severe postoperative PH, the pulmonary arterial pressure and PVR decreased without PH-targeting drugs, when the shunt circulation had been closed. This indicates that the pulmonary vascular smooth muscle remodeling was reversible in all children that survived. This notion does not contradict the findings by Miller et al. In their randomized, double-blind study on the use of inhaled nitric oxide, they noticed that inhaled NO decreased PVR and seemed to decrease the risk for PH crisis during the duration of treatment [16]. However, they defined PH crisis as a pulmonary arterial/aortic blood pressure ratio ≥ 0.75, which implies that all our patients suffered from PH crisis. Interestingly, they found no significant difference in mortality, more children who received inhaled nitric oxide died (*n* = 5), than in the placebo group (*n* = 3).

In another randomized double-blinded study on the use of inhaled nitric oxide, Day et al. found no improvement on pulmonary hemodynamics, gas exchange, or incidence of PH crisis by the use of prophylactic inhaled nitric oxide compared to placebo in children after surgery for congenital heart disease [15]. In a retrospective study on children with AVSD by Journois et al. [20] they compared a historical control group with pulmonary arterial/aortic blood pressure ratio ≥ 0.70 who did not receive inhaled NO (*n* = 39) with a group with similar PH who received inhaled NO (*n* = 25). They noticed an overall mortality of 27 children in the analyzed group of 64 children, with a reduction in mortality from 56 to 24% in children who inhaled NO. Since we had an overall mortality of 2 children in 85 children with AVSD (2.4%) during the period with a very restrictive use of inhaled NO there seem to be other cofactors that contributed to their high mortality [21]. Gilbert et al. [6] used bosentan in three children after closure of intra-cardiac shunts with PH and showed that pulmonary arterial pressure decreased. Since this was not a randomized, blinded trial it is not possible to know if this was the natural postoperative course of remodeling of the pulmonary arteries as we found in our cohort of children with severe PH. However, the use of PH-targeting drugs seems not to affect the clinical outcome, which is in agreement with our findings. However, PH-dilating drugs may increase the risk of adverse effects by decreasing PVR and exposing the pulmonary capillaries to a high pulmonary arterial pressure with a risk of interstitial pulmonary edema.

PH crisis did occur in the PICU, but in our cohort it was exclusively related to left ventricular failure or other causes of increase in pulmonary post-capillary pressure, like unbalanced ventricles, mitral regurgitation, or pulmonary venous stenosis. There were no PH crises in the children with severe PH after the right ventricular function had recovered and they had left the PICU. Our data indicate that the occurrence of PH crisis should include the evaluation of possible left-sided problems. Administration of pulmonary vasodilating drugs should be used with caution or is contraindicated in the case of left-sided obstructive lesions with pulmonary venous congestion, such as in all our children with PH crisis. In addition, our data indicate that PH should not be an indication to directly initiate pulmonary vasodilating treatment routinely when going off bypass without excluding, for example, a high left atrial pressure causing pulmonary post-capillary hypertension [22]. In this phase, it is common to have a variable degree of left ventricular dysfunction caused by, for example, the cardioplegia or air embolism in the coronary vessels. The best treatment might be to strengthen the left ventricular function with inotropes and try to increase systemic blood pressure with vasopressors, to achieve a systemic arterial blood pressure above right ventricular pressure to guarantee blood flow to the right ventricle. The restraint from pulmonary vasodilation in children with pulmonary venous congestion indirectly protects the pulmonary capillaries from the high pulmonary arterial pressure and decreases the risk of inducing an interstitial pulmonary edema, which actually can deteriorate oxygen diffusion, impair oxygen saturation, and in the long run paradoxically maintain a high PVR. The uncritical use of pulmonary vasodilator therapy may even be harmful after closure of shunt defects, since it may postpone diagnosis of underlying post-capillary problems and worsen oxygenation [23].

This is in contrast with Taylor et al. who recommended clinicians to consider the institution of aggressive pulmonary vasodilating treatment, if pulmonary arterial/aortic blood pressure ratio ≥ 0.50 [5]. Their argument that PH crises could also arise in children with sub-pulmonary arterial pressures as the sedation was weaned could not be verified by us despite monitoring the pulmonary arterial pressure continuously. The prophylactic use of sildenafil after closure of VSD and complete AVSD influenced neither the clinical course nor the occurrence of PH crisis in the prospective randomized study by Heschl et al. [23]. On the contrary, it seems that general use of pulmonary vasodilating therapy could easily escalate in children with concealed pulmonary venous hypertension with adverse effects. The liberal use of pulmonary vasodilating treatment is also contrary to the advice and the safety warning by the FDA when they recommended against the use of sildenafil at any dose in children with PH after their review of the STARTS trial [7].

Our findings also indicate that children with intra-cardiac shunts (ASD, VSD, and AVSD) may be correctable as long as the shunt in the interventricular septum has not reversed to a continuous right-to-left shunt and a definitive continuous desaturation has not occurred as in Eisenmenger’s syndrome. All children with left-to-right shunts and signs of equilibrated ventricular pressure between the right and the left side without hypoplastic left ventricle, mitral stenosis, or pulmonary venous stenosis were corrected successfully.

The postoperative routines in the PICU during the era of cardiac correction are almost similar to what we use today and included the use of opiates (ketobemidone) and midazolam. Today we use dexmedetomidine instead of midazolam. No neuromuscular- or α-blockade were used. The median time for mechanical ventilation was 0.6 days (range 0–87.0 days) in the whole cohort (*n* = 1349) of children who underwent cardiothoracic surgery. As could be expected, children who had severe PH needed longer mechanical ventilation (median time 7.7 days, range 0.7 – 45.6 days). These children received prolonged inotropic support, sedation, fentanyl, and mechanical ventilation until pulmonary arterial pressure and clinical course were controlled and echocardiographic studies indicated that the myocardial function of the right ventricle had recovered. Episodes of PH crisis were treated with intravenous fentanyl boluses (5–10 µg/kg) and hyperventilation with 100% oxygen. The overall perioperative mortality within 30 days of surgery in the total cohort of 1349 children who underwent cardiac surgery during the study period was 1.6% (*n* = 22). None of our postoperative patients with severe PH or PH crisis died within 30 days of the correction.

The successful correction of children with bidirectional shunt flows involves assessing whether a heart catheterization is needed for determination of PVR is really necessary. The decision to perform a catheterization demands anesthesia or at least sedation, which in this category of small children with PH is associated with a significant risk of major cardiovascular complications, such as cardiac arrest, PH crisis, and death [24–27]. Catheterization in children with PH has more than 10 times the risk of major complications compared with catheterization in children without PH [25]. If a major complication occurs in association with anesthesia, there is a much better ability to initiate ECMO therapy in the surgical theater than in the catheterization laboratory. The measurement of pulmonary blood flow with indirect calorimetry is inaccurate [28] and the estimation of the left atrial pressure by pulmonary capillary wedge pressure in association with PH can be difficult and inaccurate [29]. This means that the transpulmonary pressure gradient can be misleading in the estimation of PVR. Cardiac output (CO) in children should be normalized to weight (kg) in small children and not to body surface area (BSA, m_2_) [30]. Normalized CO to BSA results in an underestimation of CO and an overestimation in calculated PVRI level. There is no association between preoperative PVR and outcome, which is in accordance with our findings [10, 11]; furthermore it does not predict the need for pulmonary vasodilators after cardiopulmonary bypass [31]. The notion that it is important to show the ability of reversibility of PVR by pulmonary vasodilating drugs has shown to be of no prognostic implication, as published in the 5th World Symposium on pulmonary hypertension in Nice, France, 2013 [11, 12]. In addition, any reversibility can be considered to be included in the clinical history and the non-invasive diagnostic evaluation by echocardiography [11]. Lastly, there has been no consensus of an established PVR that absolutely contraindicates surgery [11]. However, the surgeons should always be careful to assess the possibility of left-sided problems.

## Limitations

There are several limitations to this study. It is a single-center, retrospective study with a rather small number of patients with mixed physiologies. The reason for the small sample size was the uncommon occurrence of children who developed severe PH, similar to or exceeding systemic arterial pressure in the PICU, which prohibited a robust statistical analysis, although the findings were surprisingly consistent. The small sample size made subgroup analysis and multiple regression risk analysis not possible, but the data suggested that pulmonary post-capillary hypertension is an important predictor of severe PH. Another limitation is that this data were from patients operated on in an earlier era before the use of PH-targeting drugs were introduced; however, this was also the strength of the study to show that the lack of these drugs did not have any major impact on the children at long-term follow-up. The use of questionnaires was also an important subject of bias, which made us seek primary data from chart reviews as a complement. Our results give some suggestion that PH-targeting drugs may not be important. This, of course, would need to be assessed in larger and prospective follow-up studies.

## Conclusion

Our long-term follow-up of a cohort of children with proven, severe PH after cardiac correction showed that the majority of children normalized their pulmonary arterial pressure without the use of supplemental treatment with PH-targeting therapies. However, children with remaining left-sided cardiac problems causing pulmonary post-capillary hypertension were an important high-risk factor for bad outcome.

Since the pulmonary arterial pressure decreased without the use of PH-dilating treatment in our cohort, it seems important to perform randomized, blinded trials to prove the impact on outcome of PH-targeting drugs before postoperative PH—regardless of severity—is accepted as an indication for routine treatment.
